# The plyometric activity as a conditioning to enhance strength and precision of the finger movements in pianists

**DOI:** 10.1038/s41598-022-26025-0

**Published:** 2022-12-23

**Authors:** Kaito Muramatsu, Takanori Oku, Shinichi Furuya

**Affiliations:** 1 Sony Computer Science Laboratories, Inc., Takanawa Muse Bldg, 3-14-13, Higashigotanda, Shinagawa-ku, Tokyo, 141-0022 Japan; 2 NeuroPiano Institute, Kyoto, Japan; 3 Sophia University, Tokyo, Japan

**Keywords:** Motor control, Sensorimotor processing

## Abstract

Stability of timing and force production in repetitive movements characterizes skillful motor behaviors such as surgery and playing musical instruments. However, even trained individuals such as musicians undergo further extensive training for the improvement of these skills. Previous studies that investigated the lower extremity movements such as jumping and sprinting demonstrated enhancement of the maximum force and rate of force development immediately after the plyometric exercises. However, it remains unknown whether the plyometric exercises enhance the stability of timing and force production of the dexterous finger movements in trained individuals. Here we address this issue by examining the effects of plyometric exercise specialized for finger movements on piano performance. We compared the training-related changes in the piano-key motion and several physiological features of the finger muscles (e.g., electromyography, rate of force development, and muscle temperature) by well-trained pianists. The conditioning demonstrated a decrease of the variation in timing and velocity of successive keystrokes, along with a concomitant increase in the rate of force development of the four fingers, but not the thumb, although there was no change in the finger muscular activities through the activity. By contrast, such a conditioning effect was not evident following a conventional repetitive piano practice. In addition, a significant increase in the forearm muscle temperature was observed specifically through performing the plyometric exercise with the fingers, implying its association with improved performance. These results indicate effectiveness of the plyometric exercises for improvement of strength, precision, and physiological efficiency of the finger movements even in expert pianists, which implicates that ways of practicing play a key role in enhancing experts’ expertise.

## Introduction

Musical performance represents one of the most skillful motor behaviors, which typically requires years of extensive musical training from childhood^1–3^. Conventional musical education and training, however, may emphasize the importance of quantity of the practice^4^ and subjective experience of trained teachers and performers, due to a lack of evidence proving effectiveness of individual ways of musical practicing^5^. In contrast, most of training and education in sports are built upon accumulated evidence through the development of sports science, which has contributed to breaking records over decades^6–8^. Following a similar perspective, musical performance requires reproducible and quantitative knowledge on the effectiveness of music education and training specialized for musicians who are required to perform highly dexterous sensorimotor skills in no way inferior to athletes^5^.

One approach to discover the optimal way of practicing is to compare effects of different ways of practicing on the sensorimotor skills. For example, a previous study examined effects of variation of the temporal structure of piano practicing on neuromuscular control of the sequential finger movements in pianists^9^. While rhythmic variation of successive piano keystrokes in practicing improved maximum rate of keystrokes and altered finger muscular activation patterns in piano playing, there was no change in the rhythmic accuracy of the keystrokes following such a differential learning. Non-invasive brain stimulation using the transcranial direct current stimulation also improved fine control of the finger movements in untrained individuals, but not in trained pianists^10^. These results highlight difficulty of improving precision of repetitive finger movements in trained pianists, although a recent study discovered a rare case of achieving it through a specialized somatosensory training with a haptic device^11^.

Plyometric exercise has been known as one established training in the field of sports, which consists of a quick succession of eccentric and concentric contractions of the targeted muscle^12^. Previous studies investigating this exercise have focused mainly on fast, powerful movements of the lower extremities, such as sprinting^13^ and vertical jumping^14^, and have revealed significant reductions of muscular fatigue due to a decrease in the duration of forceful contraction compared to resistance exercises that maximally stretch the muscle spindles to the same degree^12^. Post-activation performance enhancement (PAPE) has been proposed as a putative physiological mechanism underlying the short-term improvement of the performance due to an increase in rate of force development (RFD) following some physical training such as not only high-intensity resistance exercises but also plyometric training^15^. However, evidence for the effectiveness of the plyometric exercises has been limited primarily to the lower extremity, with only a few studies in the upper extremity such as the shoulder^16^, but none in the forearm and hand that are different from the lower extremity in terms of neurophysiological and biomechanical architectures. Also, it has not been known whether the plyometric exercises enhance fine motor control (i.e. temporal accuracy and agility of finger movement) of trained individuals such as musicians. However, the increased force production capacity by the plyometric-like exercise let us postulate enhancement of precision of force production, due to a negative relationship between the force production capacity and signal-dependent noise in the motor commands^17^. In addition, a previous study also reported enhancement of movement accuracy through the plyometric training in archery player^18^.

The goal of the present study is to address effects of plyometric exercises on dexterous finger movements while trained pianists play the piano. To this aim, we assessed the time-varying trajectory of the vertical position of the piano keys, key-depression force, and finger muscular activities during the repetitive keypresses before and after the conditioning, based on previous findings of the relationship of pianistic skills with force exertion patterns^19,20^ and muscular activities^21^. Since it has been pointed out that changes in performance due to PAPE are supported mainly by elevation of muscle temperature^15^, and since its time course has been shown to accompany changes in motor skill^22,23^, muscle temperature was measured throughout the course of time before, during, and after the conditioning in this study. While several studies have investigated physiological mechanisms of piano performance and practicing^21,24,25^, there has been no study assessing the skin and muscular temperature of the finger muscles during piano practice. The present research will therefore provide performers and instructors with a basis for the application of evidence-based practice methods and conditioning regimes.

## Methods

### Participants

Twenty-six pianists participated in the experiment (Nineteen females; 18–30 yr old). All of them had undergone intensive piano training and formal musical education at music conservatories and/or privately for > 14 yr. The pianists were randomly classified into two groups (i.e. thirteen pianists in each group in a gender-matched and age-matched manner) undergoing different conditioning tasks (see details in “Experimental tasks” section). While previous studies that investigated the effect of plyometric exercise on the lower-extremity changed the training protocol between males and females due to a difference in muscle strength^12^, the present study did not make any changes in the training protocol between them, because the fingertip exercise does not require large force production as compared to the lower extremity movements. In accordance with the Declaration of Helsinki, the experimental procedures were explained to all participants. Informed consent was obtained from all participants prior to participating in the experiment. All procedures were approved by the ethics committee at the Sony Corporation.

### Experimental setup

A digital piano with a real key action (KAWAI, VPC-1) was used in the experiment to collect data representing the timing, pitch, and velocity of the individual key presses and releases (i.e. MIDI information) with a custom-made LabVIEW (National Instruments) program. The instrumental sound was elicited via a headphone attached on the participant’s ears. The surface electromyography (EMG) system with two sets of wirelessly connected electrodes (Trigno Quattro sensors, Delsys Inc.) was connected to a laptop through an analog-to-digital board (NI USB-6363; National Instruments). Each electrode was placed on the muscle belly of the extensor digitorum communis (EDC) and flexor digitorum superficialis (FDS) of the right hand. The EMG signals were amplified, band-pass filtered (10–500 Hz), and sampled at 1 kHz using LabVIEW. As with EMG, a custom-made force sensor connected to an analog-to-digital convertor was used to measure the force when each finger was pressing down the sensor. A high resolution position sensor system was mounted on the bottom of the key-bed^26^, and the vertical position of the keys was recorded by 1 kHz in synchronization with MIDI and EMG. The muscle temperature of the FDS was measured at each time point throughout the experiment (see Fig. 6) with a time resolution of 500 ms using the 3 M™ Bair Hugger™ Temperature Monitoring System. Skin temperature nearby FDS was measured at each time point by infrared thermometer non-contact body temperature measurement device (easytem HPC-01; HARASAWA PHARMACEUTICAL Co., ltd). Participants were instructed to avoid having any exercises prior to the experiment and familiarization with the piano before the task.

### Experimental tasks

The experiment consisted of three successive sessions within a single day: pre-test, conditioning, and post-test. In addition, the post-test session consisted of three trials with a break in between; 0 min, 10 min, and 25 min after the conditioning, so as firstly to assess the effects of conditioning and its retention, and secondly to infer whether PAPE or PAP are candidate physiological mechanisms of the present conditioning^15^. In the pre-test and post-test sessions, participants were asked to perform two tasks: the piano test and finger force production test. At the beginning of the experiment, the isometric maximal voluntary contraction (MVC) was asked to be performed at the EDC and FDS to calculate %MVC during the task performance in a manual maneuver. Participants pressed down the four adjacent keys of the keyboard with all 2–5 fingers simultaneously for 1 s as strong as possible for assessing the MVC of FDS. During the measurement of MVC of EDC, participants pushed up the experimenter’s hand that was put on the participant’s hand with the back of all of the 2–5 fingers (i.e. extending the fingers) simultaneously as strong as possible for 1 s. The MVC assessment was performed twice for each of these two muscles. The maximum amplitude of the root-mean-squared EMG signal was calculated for each trial, and the larger value among the two was adopted as MVC.

For the piano test, participants played the melody designated on a test score (see Fig. 2A right) with the piano by the right hand, while their elbow was put on a table to minimize motions of the other body portions (i.e. forearm only). Furthermore, they were instructed so that the fingers could be kept on the surface of the keyboard from the beginning of the keystroke as much as possible and that the wrist could be immobilized without any rotational movements. The participants were asked to play with maintaining the tempo of 100 beats per minute (BPM, i.e. with the inter-tone duration of 100 ms due to sextuplet) as accurately as possible during the task performance with keeping the loudness as consistent as possible. The tempo was provided with a metronome only before each performance was initiated.

For the finger force production test, the elbow and wrist were immobilized on a table, and only the fingers were used to press the force sensor in a manner displayed on the musical score (see Fig. 4A). As in the piano test, their fingertips were always kept in contact with the force sensors. Then the participants were asked to press as strongly and quickly as possible isometrically, along with the tempo provided by the metronome. The maximum pressure to be exerted during this maximum isometric force production was regarded as RFD, because RFD has been approximated as such at the instantaneous maximum-force production^15^ as short as one used in the present study.

**Figure 1:**
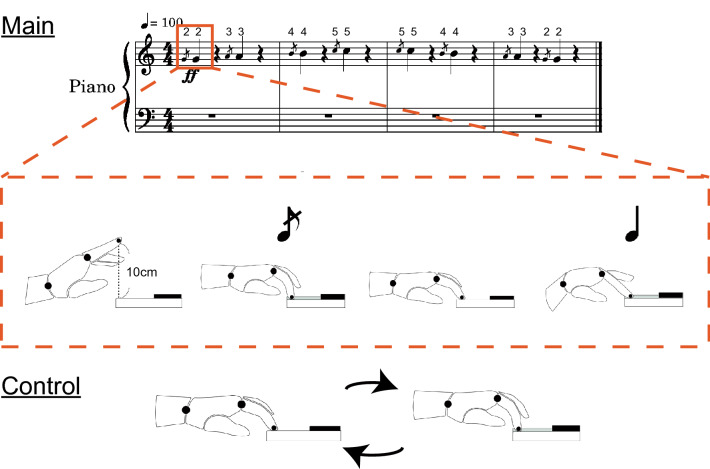
A musical score representing the conditioning task (top panel) and a schematic drawing of the keystroke movements corresponding to the repetitive keystrokes used by the main group with the plyometric activity (middle panel) and control group who underwent the same number of keystrokes as the main group (bottom panel). The number on the score represents the fingering (2, 3, 4, and 5 corresponds the index, middle, ring, and little finger, respectively).

In the conditioning session, participants were asked to perform the conditioning task with the right hand in an instructed manner that differed between the groups. Participants in the main group were instructed to perform a plyometric-like exercise with the piano 40 times in a manner displayed on the score (see the score in Fig. 1), which was characterized as follows: (1) one continuous cycle of swinging the hand down from 100 mm above the keyboard toward the key and returning to the original position, (2) two strong strikes in succession as a unit, the first with the downward elbow motion and the second with the wrist flexion (i.e. snapping), (3) being aware of relaxing the muscles except at the moment of each enunciation, (4) making the interval between two successive strikes as short as possible. By contrast, participants in the control group repeated the same exercise as the aforementioned piano test 40 times, so that the total duration of the conditioning session could be the same between the main and control groups. In both of the groups, 40 time repetitions were divided by 4 sets (i.e. 10 time exercises per a set).

### Data analysis

#### Movement variables

The MIDI information obtained from the keyboard was used as variables for evaluating the keystroke performance. In order to minimize the effect of individual differences in the average tempo and loudness, the coefficient of variation (CV) of the inter-keystroke interval (IKI) of two successive strikes was used as a variable representing stability of the tempo, whereas the CV of the keystroke velocity was used as an index representing the loudness stability^27^.

Data of the finger pressure and key motions were cut for each press/keystroke as epochs according to a threshold (three times of the standard deviation of the signals prior to the task performance), which was used for time normalization of the epochs. These data were averaged across the epochs for smoothing, and the diff function in MATLAB (Mathworks Inc.) was used to compute the first and second derivatives of the vertical position of the key. The maximum value of each waveform was used for the subsequent analyses.

#### EMG preprocessing

The EMG data were bandpass filtered at 10–250 Hz in an offline manner to remove artificial high-frequency noise and movement artifacts with MATLAB using signal processing toolbox. The same time-index was used for time normalization of the EMG signals to temporally align each epoch with the time normalized force and key motion.

### Statistics

A two-way mixed-design ANOVA (independent variables: Group and Condition) or three-way mixed-design ANOVA (independent variables: Group, Condition, and Finger) was run as needed using ez package in R (open source). Here, “Condition” has four levels (i.e. pre-test and three post-tests: 0 min, 10 min, and 25 min), whereas “Group” has two levels (i.e. two different sets of training). If Mauchly's sphericity test was necessary, the Greenhouse–Geisser correction was performed. Post-hoc tests were performed using Benjamini and Hochberg correction for multiple comparisons^28^ only in the case of significance with correction for multiple comparisons (p < 0.05).

## Results

### Conditioning effects on variability of the inter-keystroke intervals and keypress velocity

**Figure 2:**
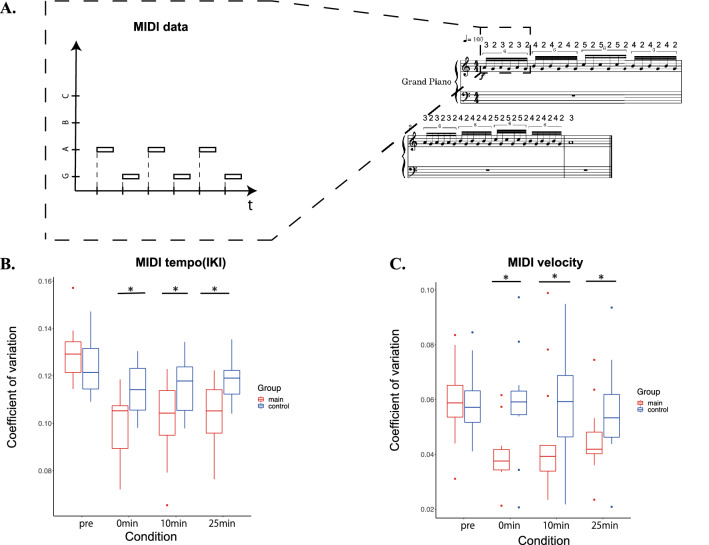
(**A**) A schematic illustration of the temporal information of the individual keystrokes representing a musical score representing the test task. (**B**) and (**C**) Box plots of the group means of the coefficients of variation (CV) of the timing (MIDI Inter-keystroke Intervals: IKI) and velocity (MIDI velocity) of the keypresses before and after the conditioning session (i.e. condition in the x-axis) in the main (red box) and control (blue box) groups. *: p < 0.05.

Figure 2 illustrates the group means of the coefficient of variation of the inter-keystroke interval (Fig. 2B) and that of the keypress velocity (Fig. 2C) at the piano test (Fig. 2A) before and after the conditioning session in the main and control groups. For the rhythmic variability of the keystrokes, a two-way mixed-design ANOVA with group and condition yielded both interaction effect (F(3,72) = 3.967, p = 1.23 $\times {10}^{-4}$, ${\upeta }^{2}$=0.053) and main effect of condition (F(3,72) = 14.51, p = $1.73\times {10}^{-7}$, ${\upeta }^{2}$ =0.170), but no main effect of group (F(1,24) = 5.19, p = 0.48, ${\upeta }^{2}$ = 0.014). Post-hoc comparison showed group differences only after the conditioning session. For the inter-strike variability of the keypress velocity, both the interaction effect between group and condition (F(3,72) = 8.736, p = 5.13 $\times {10}^{-5}$, ${\upeta }^{2}$= 0.048) and main effect of condition (F(3,72) = 11.58, p = 2.80 $\times {10}^{-6}$, ${\upeta }^{2}$= 0.062) were significant, whereas there was no main effect for group (F(1,24) = 3.029, p = 0.095, ${\upeta }^{2}$= 0.098). Post-hoc analysis was also performed to test within-group changes from pre-conditioning to post-conditioning for the control and main groups. Neither the variability of IKI and MIDI velocity was significantly different between the pre-conditioning and 0 min conditions in the control group (p__IKI_ = 0.321, p__vel_ = 0.976), whereas those in the main group was significantly decreased from the pre-conditioning to the 0 min condition (p__IKI_ = 0.002, p__vell_ = 0.027).

### Effects of conditioning activity on the piano key-descending velocity and acceleration

**Figure 3:**
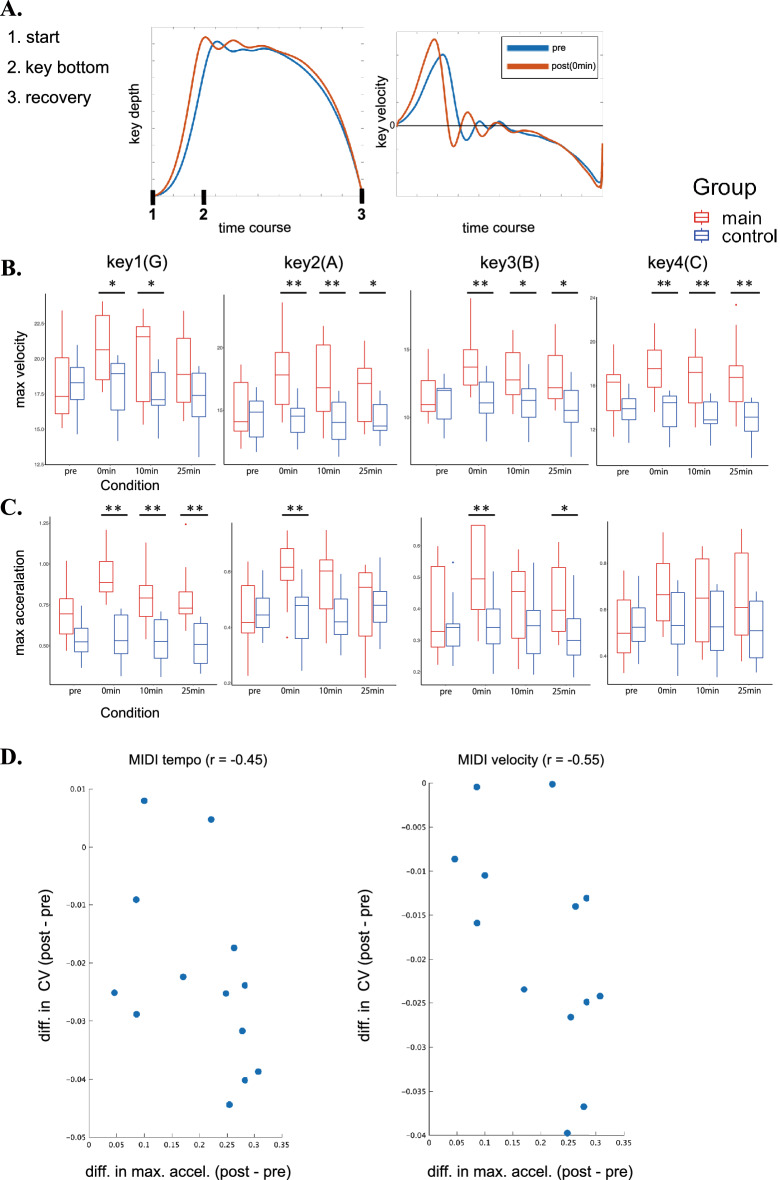
(**A**) Representative examples of the time-varying trajectories of the vertical position of the piano key (left) and their derivatives (right) at the pre-test (blue) and post-test (i.e. 0 min after the conditioning) (red) of one representative pianist in the main group. (**B**) and (**C**) Box plots of the group means of the maximum descending velocity (**B**) and acceleration (**C**) of the trajectories of the four keys to be struck (i.e. key1–4) before and after training (x-axis) in the main (red box) and control (blue box) groups. *p < 0.05, **p < 0.01. (**D**) Scatter plots of the differential values between the pre-test and post-test in the maximum keystroke acceleration relative to the CV of the keystroke timing (left panel) and velocity (right panel).

Figure 3 shows the group means of the maximum descending velocity (Fig. 3B) and acceleration (Fig. 3C) of the key-motion at the piano test (Fig. 2A) before and after the conditioning session in the main and control groups. For the maximum key velocity, a two-way mixed-design ANOVA with group and condition revealed a significant interaction effect as well as main effects of both group and condition for all keys (see Table 1). Post-hoc comparison did not show any group differences before the conditioning (i.e. pre-test). On the other hand, there were significant group differences after the conditioning session (i.e. 0 min, 10 min, 25 min), for all keys. For the maximum acceleration, the interaction effects were evident at all of the four keys to be struck, whereas the main effects of the group at the key-1 and key-3 and the main effects of condition at all keys were significant (see Table 2). Post-hoc comparison yielded no group differences before the conditioning (i.e. pre-test). On the other hand, there were significant group differences after the conditioning session (i.e. 0 min, 10 min, 25 min) for all keys except for the key-4. We also found a negative correlation of the differential value of the maximum acceleration of the key descending motion between the pre-test and post-test in the main group, both with the variability of the inter-keystroke intervals (r = − 0.45) and that of the keypress velocity (r = − 0.55), respectively.

**Table 1:** Results of two-way mixed-design ANOVA for the maximum velocity and acceleration of the key-motion.

Variable		Fixed effect								
		Group			Condition			Group × condition		
		F	P	η^2^	F	P	η^2^	F	P	η^2^
**Key motion**										
Key 1	Max-velocity	**4.732**	**3.97 × 10**^**–2**^	**0.156**	**14.24**	**1.36 × 10**^**–6**^	**0.036**	**19.7**	**2.36 × 10**^**–8**^	**0.049**
	Max-acceleration	**23.97**	**5.41 × 10**^**–5**^	**0.472**	**17.76**	**9.96 × 10**^**–9**^	**0.07**	**12.25**	**1.45 × 10**^**–6**^	**0.049**
Key 2	Max-velocity	**9.632**	**4.85 × 10**^**–3**^	**0.267**	**16.85**	**2.16 × 10**^**–8**^	**0.058**	**19.73**	**1.94 × 10**^**–9**^	**0.068**
	Max-acceleration	2.198	1.51 × 10^–1^	0.071	**13.78**	**3.42 × 10**^**–7**^	**0.068**	**23.24**	**1.25 × 10**^**–10**^	**0.109**
Key 3	Max-velocity	**7.152**	**1.33 × 10**^**–2**^	**0.218**	**26.27**	**1.38 × 10**^**–11**^	**0.062**	**21.9**	**3.49 × 10**^**–10**^	**0.052**
	Max-acceleration	**6.825**	**1.53 × 10**^**–2**^	**0.201**	**13.02**	**6.96 × 10**^**–7**^	**0.037**	**14.46**	**1.82 × 10**^**–7**^	**0.041**
Key 4	Max-velocity	**14.17**	**9.54 × 10**^**–4**^	**0.356**	**8.113**	**9.99 × 10**^**–5**^	**0.021**	**15.04**	**1.07 × 10**^**–7**^	**0.038**
	Max-acceleration	3.242	8.43 × 10^–2^	0.109	**10.71**	**6.68 × 10**^**–6**^	**0.041**	**8.375**	**7.54 × 10**^**–5**^	**0.032**

Significant values are in bold.

**Table 2:** Results of two-way mixed-design ANOVA for each of the finger pressure and activations of the EDC (extensor) and FDS (flexor) muscles in the finger force production task.

Variable	Fixed effect																				
	Group			Condition			Finger			Group × condition			Group × finger			Condition × finger			Group × condition × finger		
	F	P	η^2^	F	P	η^2^	F	P	η^2^	F	P	η^2^	F	P	η^2^	F	P	η^2^	F	P	η^2^
**Finger pressure**																					
Max	**5.66**	**2.56 × 10**^**–2**^	**0.139**	**25.39**	**4.73 × 10**^**–9**^	**0.035**	**13.39**	**7.75 × 10**^**–8**^	**0.109**	**18.91**	**2.10 × 10**^**–7**^	**0.026**	1.1	3.57 × 10^–1^	0.01	**7.42**	**1.68 × 10**^**–9**^	**0.019**	1.85	6.01 × 10^–2^	0.005
Time to peak	0.297	5.91 × 10^–1^	0.006	0.272	0.781	0.002	**3.702**	**0.0454**	**0.016**	0.716	0.505	0.006	0.356	0.645	0.002	0.988	386	0.007	1.464	0.24	0.011
**EMG (finger force development task)**																					
Max (EDC)	0.099	1.42 × 10^–1^	0.012	0.737	2.22 × 10^–1^	0.006	0.626	2.70 × 10^–1^	0.015	0.415	7.43 × 10^–1^	0.001	1.388	2.44 × 10^–1^	0.002	0.711	7.40 × 10^–1^	0.002	0.999	4.50 × 10^–1^	0.003
Max (FDS)	2.446	1.31 × 10^–1^	0.063	0.841	2.79 × 10^–1^	0.004	0.948	2.69 × 10^–1^	0.007	1.77	8.77 × 10^–2^	0.003	0.974	4.25 × 10^–1^	0.009	0.677	4.43 × 10^–1^	0.002	1.05	3.97 × 10^–1^	0.005
Interval of peaks	1.143	2.96 × 10^–1^	0.018	0.124	9.45 × 10^–1^	0.0001	0.774	5.12 × 10^–1^	0.036	0.755	5.23 × 10^–1^	0.001	2.221	9.26 × 10^–1^	0.004	1.481	1.31 × 10^–1^	0.007	2.883	9.91 × 10^–1^	0.001

Significant values are in bold.

### Effects on the maximum finger force exertion

**Figure 4:**
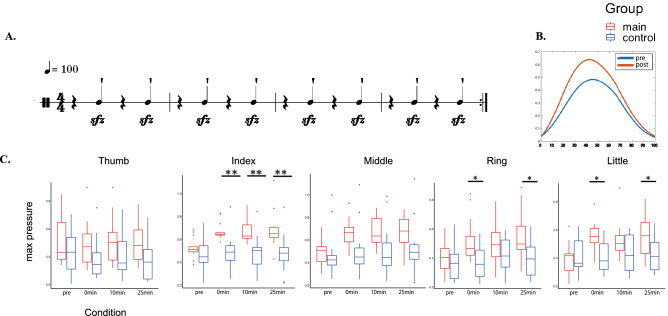
(**A**) A score in the finger force production test. (**B**) Representative trajectories of the finger pressure of one pianist. C: Group means of the maximum pressure exerted by each of the five digits before and after the conditioning task (x-axis; condition) in the main (red) and control (blue) groups. *p < 0.05, **p < 0.01.

In order to identify factors associated with the aforementioned results, we investigated effects of the plyometric activity on RFD during the finger force exertion in the finger force production test. Figure 4 shows the group means of the maximum finger force exerted by each of the four fingers (Fig. 4C) at the designated finger force production task (Fig. 4A) before and after the plyometric activity in the main and control groups. A three-way mixed-design ANOVA with group, condition, and finger was performed for the maximum exerted force (see Table 2). There was no second-order interaction, whereas significant first-order interactions were found for both Finger × Condition and Group × Condition, but not for Group × Finger. The main effects of all three factors were also significant. For each finger, post-hoc comparison was conducted for Group × Condition, and overall groupwise differences were evident for the fingers 2, 4, and 5, but not for the fingers 1 and 3. For the time to which the exerted finger force reached its peak value, ANOVA revealed the main effect only of the finger, but none of the interactions nor the other main effects were significant (Table 2).

### Finger muscular activities during the finger force production test

**Figure 5:**
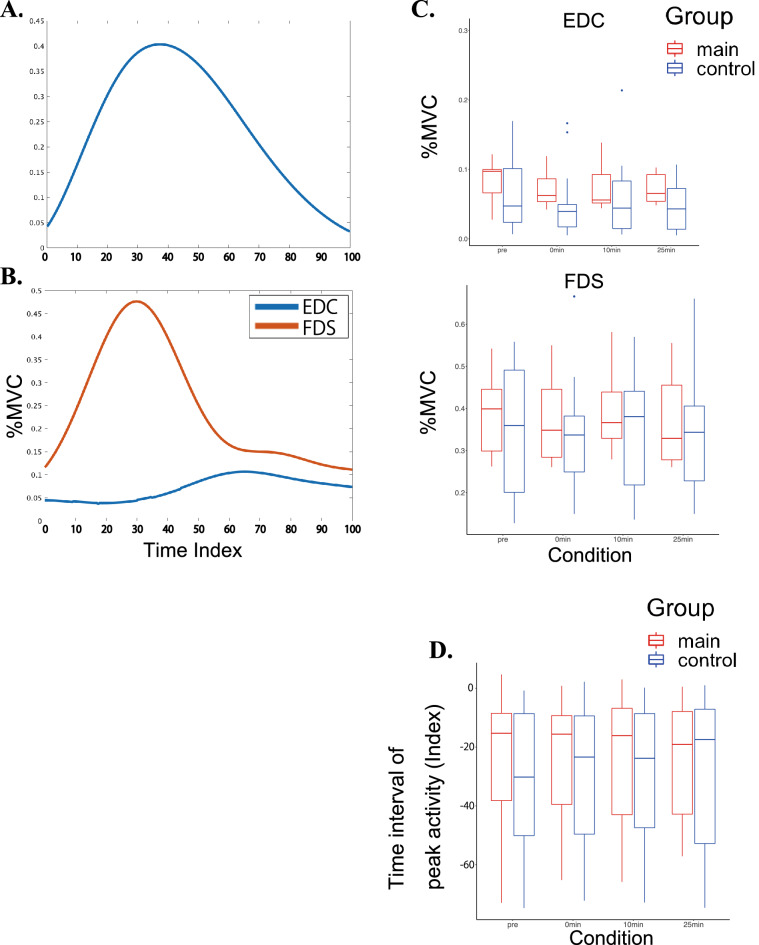
(**A**) and (**B**) Representative examples of the time-varying trajectory of the finger pressing force (**A**) and its corresponding muscular activities of the finger extensor and flexor muscles (i.e. EDC and FDS) (**B**) of one representative pianist in the main group The x-axis indicates the normalized time so that the period from the initiation to the termination of the force production can be 100 timepoints. (**C**) Box plots of group means o the maximum values of the muscular activities at the EDC and FDS before and after the conditioning session in the main (red) and control (blue) groups. (**D**) Box plots of group means of the interval of the timing of the peak activities between EDC and FDS in the main and control groups. The negative value indicates when the peak FDS activity preceded the peak EDC activity. *p < 0.05, **p < 0.01.

Figure 5 illustrates the group means of the maximum activities of the EDC and FDS muscles (Fig. 5C) and the time-varying waveforms of these muscular activities (Fig. 5B) along with the force exerted by the index finger (Fig. 5A) during the finger force production test. For the maximum values, a three-way mixed-design ANOVA with group, condition, and finger showed that neither the interactions nor main effects were significant for each of the EDC and FDS (Table 2). Similarly, for the time interval of the peak activities between the EDC and FDS (Fig. 5D), a three-way mixed-design ANOVA yielded neither significant interaction nor main effects.

### Effects of plyometric activity on changes of muscle and skin temperature

**Figure 6:**
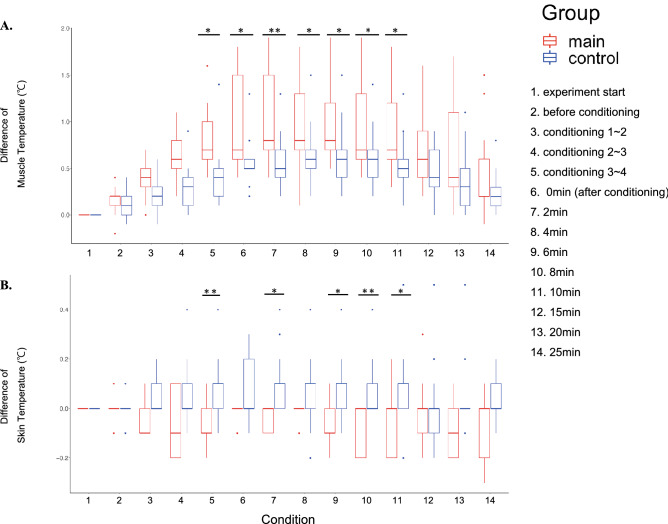
A time-course of the temperature of the finger flexor muscle at the forearm (**A**) and skin of the forearm (**B**) throughout a course of the experiment in the main group (with the plyometric activity in red) and control group (without the plyometric activity in blue). The x-axis indicates the conditions, each of which is indicated at the right box in the figure. *p < 0.05, **p < 0.01.

Figure 6 shows the group means of the time-varying muscle temperatures at FDS (Fig. 6A) and the forearm skin temperatures (Fig. 6B) throughout the experiment. A two-way mixed-design ANOVA with condition and group was performed for the muscle temperature and found a significant interaction(F(13,312) = 3.154, p = 4.23 $\times {10}^{-2}$, ${\upeta }^{2}$=0.032), main effects of group (F(1,24) = 5.035, p = 3.43 $\times {10}^{-2}$ , ${\upeta }^{2}$= 0.136) and condition (F(13,312) = 53.48, p = 6.71 $\times {10}^{-15}$, ${\upeta }^{2}$= 0.359). Post-hoc comparisons revealed group differences particularly during the period from the second half of the conditioning to 10 min after the conditioning. A two-way mixed-design ANOVA for the skin temperature similarly showed both a significant interaction (F(13,312) = 2.602, p = 1.93 $\times {10}^{-2}$, ${\upeta }^{2}$=0.049) and main effect of group (F(1,24) = 9.968, p = 4.26 $\times {10}^{-3}$, ${\upeta }^{2}$ =0.179), but no condition effect (F(13,312) = 1.364, p = 2.32 $\times {10}^{-1}$, ${\upeta }^{2}$=0.026). Post-hoc comparisons showed significant group differences during the period similar to that of the muscle temperature.

## Discussion

The present study found that the plyometric conditioning targeting the finger flexor muscle was effective as exemplified by a decrease of the variability of both timing and velocity of the keystrokes when performing a pianistic task that requires loud and fast tone production. On the other hand, such a significant effect of the conditioning on the accuracy of movement was not observed following the activity with repetitive piano keystrokes that did not involve the plyometric-like muscular contraction (i.e. a control group). The contrasting group difference indicates that the plyometric exercises used in this study enhances precision of the finger movements in fast and forceful repetitive piano keystrokes, which has been difficult to be achieved in previous studies. Interestingly, the spatiotemporal features of the finger muscular activities did not change following the plyometric exercise for both FDS and EDC, whereas the finger muscular but not skin temperature was elevated as the plyometric exercise was being performed. This suggests physiological changes at the finger muscles by the plyometric activity. Together, these results indicate that the plyometric exercise has potentials of further improving well-trained performance skills of pianists who underwent extensive training and have difficulty of improving their skill through conventional training (i.e. ceiling effect)^9–11^.

To evaluate the conditioning effect on the finger motor functions, we assessed RFD in the isometric finger force production for flexion. Specifically following the plyometric-like piano exercise, RFD was increased for each of the four fingers that underwent the activity. A lack of any conditioning effect at the thumb that did not perform the plyometric conditioning and at all fingers that underwent the conventional repetitive practicing supports the idea that the enhanced ability of the finger force production resulted from this plyometric activity. Similarly, the conditioning effect was also evident for the maximum acceleration of the piano key-depression, which was correlated with improvement of precision of timing and velocity in the piano keystrokes. One possible explanation for the enhanced piano performance is a negative physiological relationship between the muscular strength and variability of the force production^17^. It is therefore plausible that the strengthening effect of the plyometric exercise on the finger muscles aided in reducing signal-dependent noise in the motor commands issued into the muscles and thereby decreased the variability of the exerted force. Interestingly, the muscular activation was not augmented through the conditioning (i.e. EMG amplitude), even immediately after the conditioning before taking rest (at 0 min) and its subsequent period following the conditioning, although the force production was increased. This indicates that the target force can be produced with reduced finger muscular activities of the finger, implicating improvement of physiological efficiency in the finger force production. This can play a role in preventing muscular fatigue and/or development of overuse syndromes through piano practicing, in addition to enhancement of timing and force precision in piano performance.

As one putative physiological mechanism behind the effect of the plyometric conditioning on the force production ability, we found elevation of the finger muscular temperature but not of the skin temperature specifically following this conditioning. Muscle contraction begins with the release of calcium ions from the sarcoplasmic reticulum into the myofibrils, which binds actin and myosin heads (cross-bridges) and then consumes energy from the ATPase reaction for contraction. Previous studies have shown that the cross-bridge cycling rates for muscle contraction are affected largely by the temperature-dependent myosin-ATPase reaction^29,30^, which explains why the muscle temperature was elevated along with the increase in RFD of the skeletal muscles in both present and previous studies^26,31,32^. In other words, the increase in RFD may be due to an increase in the chemical reaction rate during muscle contraction as the muscle temperature elevates, which has been observed as an increase in the muscle power output in passively warming (water immersion, ~ 1 °C) hands^33^. This can be a potential reason why the RFD was increased through the plyometric activity.

The phenomenon of improved motor performance following a conditioning exercise is called PAPE, which is recently proposed to distinguish it from conventional Post Activation Potentiation (PAP) due to Myosin Light Chain Phosphorylation^11^. These two phenomenon are considered to be different with respect to their temporal dynamics (i.e. PAP: ~ 5 min, PAPE: 15 min ~) and to muscle temperature change (i.e. only PAPE but not PAP accompanies it)^15^. It has been shown that plyometric exercise can induce PAPE as a conditioning exercise not only in the lower limb but also in the upper limb at the short force production rather than resistance exercises with high and lasting long force production^12,34^. In the present study, we used plyometric conditioning for the finger muscle, which demonstrated concomitant changes in the finger muscular temperature along with the plyometric-related activity, and eventually an increase in the RFD at the four fingers that underwent the conditioning. Also, the increase in RFD and motor performance lasted 25 min, which also supports PAPE. Several remarkable effects of skill improvement by PAPE has been demonstrated in tasks especially with high-speed force exertion such as sprinting and jumping. Piano performance in this study similarly require high-speed movements and strong muscle contractions of more than 10 times per second, and the results indicate that the application of PAPE is highly compatible with such movements. This observation suggests that selective application of plyometric exercises to the finger extrinsic muscles induced PAPE, which may underlie the improved motor skills in piano performance. Several alternative explanations for the skill improvement still remain, which include that motor learning with a modified rhythm of the target task optimized muscle coordination of performance as was observed in a previous study^5^, or that participants acquired a different way of keystrokes toward approaching the key surface through the activity, despite instructions to keep the fingers contact with the key throughout the performance.

There are several limitations in the present study. First, to infer the physiological mechanism of the present conditioning effect, it is necessary to assess chemical changes within the muscles through the activity and neuroplastic changes in the primary motor cortex and spinal cord by means of non-invasive brain stimulation techniques. In future studies, it is also necessary to record activities of the intrinsic hand muscles and the other extrinsic muscles in order to uncover the entire physiological mechanism of the plyometric activity. Second, it is necessary to evaluate whether the plyometric activity effect on the finger movements has any gender-wise difference, because previous studies investigating the plyometric conditioning with the lower extremity found a differential effect between males and females, which should be also tested with the upper extremity. Third, the effect of different protocol and/or intensity of the plyometric conditioning should be systematically investigated (e.g. the number of repetitions, movement amplitude, days of training) in order to optimize the conditioning effect. Fourth, although the present study focused on PAPE as a putative mechanism of effects of the plyometric exercise on the motor performance, other candidate mechanisms should be also studied such as changes in the intracortical functions of the motor cortex and muscle condition such as muscle water contents or degree of fatigue. Last but not the least, the present study did not fully validate the application of plyometric activity to the finger due to a lack of any previous study of its application to the same body portion. However, we found indirect evidence of PAPE through the measurement of muscle temperature, which can be considered as an indirect physiological evidence of the plyometric activity.

## Data Availability

The datasets used and/or analysed during the current study available from the corresponding author on reasonable request.
